# *Aspergillus felis* in Patient with Chronic Granulomatous Disease

**DOI:** 10.3201/eid2512.191020

**Published:** 2019-12

**Authors:** Olivier Paccoud, Romain Guery, Sylvain Poirée, Grégory Jouvion, Marie Elisabeth Bougnoux, Emilie Catherinot, Olivier Hermine, Olivier Lortholary, Fanny Lanternier

**Affiliations:** Hôpital Necker-Enfants Malades, Paris, France (O. Paccoud, R. Guery, S. Poirée, M.E. Bougnoux, O. Hermine, O. Lortholary, F. Lanternier);; Institut Pasteur, Paris (G. Jouvion, O. Lortholary, F. Lanternier);; Hôpital Foch, Université Versailles-Saint-Quentin-en-Yvelines, Versailles, France (E. Catherinot)

**Keywords:** *Aspergillus felis*, invasive pulmonary aspergillosis, allergic bronchopulmonary aspergillosis, chronic granulomatous disease, fungi

## Abstract

We report a case of *Aspergillus felis* infection in a patient with chronic granulomatous disease who had overlapping features of invasive pulmonary aspergillosis and allergic bronchopulmonary aspergillosis. Identifying the species responsible for aspergillosis by molecular methods can be crucial for directing patient management and selection of appropriate antifungal agents.

A 42-year-old man with X-linked chronic granulomatous disease (CGD) sought care at a hospital in Paris, France, for a 2-week history of cough and night sweats. He had been receiving long-term prophylaxis with itraconazole (400 mg/d) and had normal trough levels (1,240 μg/L) 1 month before his hospital visit. 

At admission, blood counts showed mild leukocytosis (leukocytes 9.6 × 10^9^ cells/L, reference range 4–10 × 10^9^ cells/L), with neutrophils at 6.1 × 10^9^ cells/L (reference range 1.5–7 × 10^9^ cells/L) and eosinophils at 2 × 10^9^ cells/L (reference <0.5 × 10^9^ cells/L). Computed tomography (CT) revealed an upper left lobe consolidation (Appendix Figure). We administered broad-spectrum antimicrobial drugs (2 g meropenem 3×/d and 20 mg/kg/d amikacin). Results of bacterial and mycological cultures from sputum were negative, as was serum galactomannan. 

The patient’s condition did not improve, so we administered liposomal amphotericin B (5 mg/kg/d) and caspofungin (70 mg/d loading dose followed by 50 mg/d). Bronchoalveolar lavage demonstrated hypercellularity (1.22 × 10^6^ cells/mL); manual differential showed 12% macrophages and 76% eosinophils. Results of bacterial, mycological, and mycobacterial cultures were negative. Pathology studies from a transbronchial biopsy revealed numerous eosinophilic granulomas alongside Charcot-Leyden crystals (Appendix Figure). Grocott methenamine silver staining revealed rare septated filamentous hyphae, but results of mycological cultures were negative. The patient had elevated total serum IgE (1,210 IU/mL, reference <114 IU/mL), elevated serum *A. fumigatus* IgE (7 IU/mL, reference <0.1 IU/mL) and *A. fumigatus* IgG (54 IU/mL, reference <5 IU/mL), and precipitating antibodies to *A. fumigatus* (2 arcs of precipitation in immunoelectrophoresis). Results of parasitologic examination of fecal samples and serologic testing for alternative causes of eosinophilia were negative.

 Eosinophilia persisted (1.8–2 × 10^9^ cells/L) despite antiparasitic treatment with ivermectin (5 mg/kg/d at days 1 and 7) and albendazole (400 mg/d for 7 d). Pathology findings from a transthoracic percutaneous biopsy revealed granulomas with Grocott-positive septated hyphae. Result of an *Aspergillus* section Fumigati PCR on a biopsy specimen were positive, and mycological cultures yielded a mold morphologically identified as *Aspergillus*. After 5 weeks of liposomal amphotericin B therapy (including 2 weeks of combination therapy with caspofungin), we switched treatment to oral voriconazole (loading dose of 400 mg 2×/d, followed by 200 mg 2×/d). Normalization of eosinophilia occurred at 6 weeks. 

We sent mycological cultures from the biopsy specimens to the French National Center for Invasive Mycoses and Antifungals (Paris). Molecular identification based on the partial sequence of the internal transcribed spacer 2, 5.8S ribosomal RNA gene, and internal transcribed spacer 2 (525/526 bp; 99% similarity to the type strain, CBS 130245; GenBank accession no. KF558318.1) and the β-tubulin target gene enabled the identification of *Aspergillus felis* (109/109 bp; 100% similarity to the type strain, CBS DTO_131-E3 β-tubulin [*benA*] gene, partial cds; GenBank accession no. KY808576.1). The European Committee for Antimicrobial Susceptibility Testing (EUCAST) MICs with broth microdilution methods (*1*) were 4 μg/L for voriconazole, 4 μg/L for itraconazole, 0.25 μg/L for posaconazole, 2 μg/L for caspofungin, and 4 μg/L for amphotericin B. Based on EUCAST MIC breakpoints for *A. fumigatus* (*2*), we switched treatment to oral posaconazole (loading dose of 300 mg 2×/d, followed by 300 mg/d). Chest CT performed 12 months after treatment initiation showed noticeable improvement of pulmonary lesions.

Invasive pulmonary aspergillosis (IPA) remains a leading cause of death during CGD and IPA typically manifests as subacute pneumonia, with little or no angioinvasion (*3*). This patient had pulmonary infection caused by *A. felis* with overlapping features of IPA and allergic bronchopulmonary aspergillosis (ABPA) (*4*). Sensitization to *Aspergillus* spp. in patients with CGD (*5*) and tissue eosinophilia in lung pathology studies during invasive fungal infections (*6*) have been reported but do not seem to be common features of IPA in patients with CGD (*3*,*7*). There was some uncertainty about whether *A. felis* was responsible for this overlapping phenotype between IPA and ABPA (Table).

**Table:** Defining features of invasive pulmonary aspergillosis and allergic bronchopulmonary aspergillosis in a 42-year-old man with X-linked chronic granulomatous disease, Paris, France*

Category	Defining features			
	IPA (oncohematologic setting)	IPA during CGD	Patient in this study	ABPA
Underlying disease	Neutropenia	CGD, particularly X-linked CGD	X-linked CGD	Asthma, cystic fibrosis
Mechanisms of disease	Angioinvasion	Tissue invasion, little or no angioinvasion	No angioinvasion	Exaggerated inflammatory response to *Aspergillus*
Course of infection	Acute, single event	Subacute or chronic, single event	Subacute, single event	Chronic with exacerbations
Radiographic findings	Cavitation, pulmonary infarction, air crescent sign, halo sign	Single or multiple nodules and consolidations	Single consolidation	Central bronchiectasis, pulmonary infiltrates, mucus plugs
Galactomannan testing	Positive	Positive or negative	Negative	Negative
Total serum IgE	Normal	Normal	Elevated (1,410 IU/L)	Elevated (>1,000 IU/L)
*Aspergillus* species-specific IgE or skin test reactivity	Negative	Negative	Positive (7 IU/mL)	Positive (>0.1 IU/mL)
*Aspergillus* IgG	Negative	Negative	Positive (54 IU/mL)	Positive (>10 IU/mL)
Precipitating antibodies to *Aspergillus*	Negative	Negative	Positive (2 arcs of precipitation)	Positive (>1 arc of precipitation)
Blood eosinophilia	Absent	Absent; reported only during “fulminant mulch pneumonia”	Present (2.2 × 10^9^ cells/L)	Present (>0.5 × 10^9^ cells/L)
First-line treatment	Antifungal treatment	Antifungal treatment	Antifungal treatment	Systemic or inhaled corticosteroids

*ABPA, allergic bronchopulmonary aspergillosis; CGD, chronic granulomatous disease; IPA, invasive pulmonary aspergillosis.

*A. felis* is a member of the *A. viridinutans* complex, a group of cryptic species belonging to *Aspergillus* section Fumigati (*8*). Such fumigati-mimetic molds are increasingly being recognized as sporadic causes of IPA (*9*). *A. felis* has been reported as a cause of sino-orbital aspergillosis in cats, but less frequently in humans (*8*). In one such case of IPA, and in the few reported cases in patients with CGD of IPA caused by the closely related *A. pseudoviridinutans* and *A. udagawae*, the course of infection was more protracted than for *A. fumigatus* infections, and dissemination occurred in a contiguous manner (*10*). Nonfumigatus *Aspergillus* spp. exhibit decreased in vitro susceptibility to commonly used antifungal drugs. Most previously reported antifungal susceptibilities from *A. felis* isolates showed high MICs for voriconazole and itraconazole but lower MICs for posaconazole (*8*). 

Because isolates may be misidentified as *A. fumigatus*, culture-based morphological identification of invasive fungal infections in CGD may sometimes be insufficient. In cases of breakthrough fungal infections, or when faced with an atypical or refractory course of infection, identification of the fungus at a species level by molecular methods appears to be critical to guiding proper patient management.

Appendix Images obtained from patient with chronic granulomatous disease who had *Aspergillus felis* infection.
