# Supplementary material for: Aspergillus felis in Patient with Chronic Granulomatous Disease
Source: Emerg Infect Dis. 2019 Dec;25(12):2319–21. doi: 10.3201/eid2512.191020 (PMC6874246; doi:10.3201/eid2512.191020)
Supplement: Appendix — Images obtained from patient with chronic granulomatous disease who had Aspergillus felis infection. [file 19-1020-Techapp-s1.pdf]

Article DOI: https://doi.org/10.3201/eid2512.191020

Aspergillus felis in Patient with Chronic Granulomatous Disease

Appendix

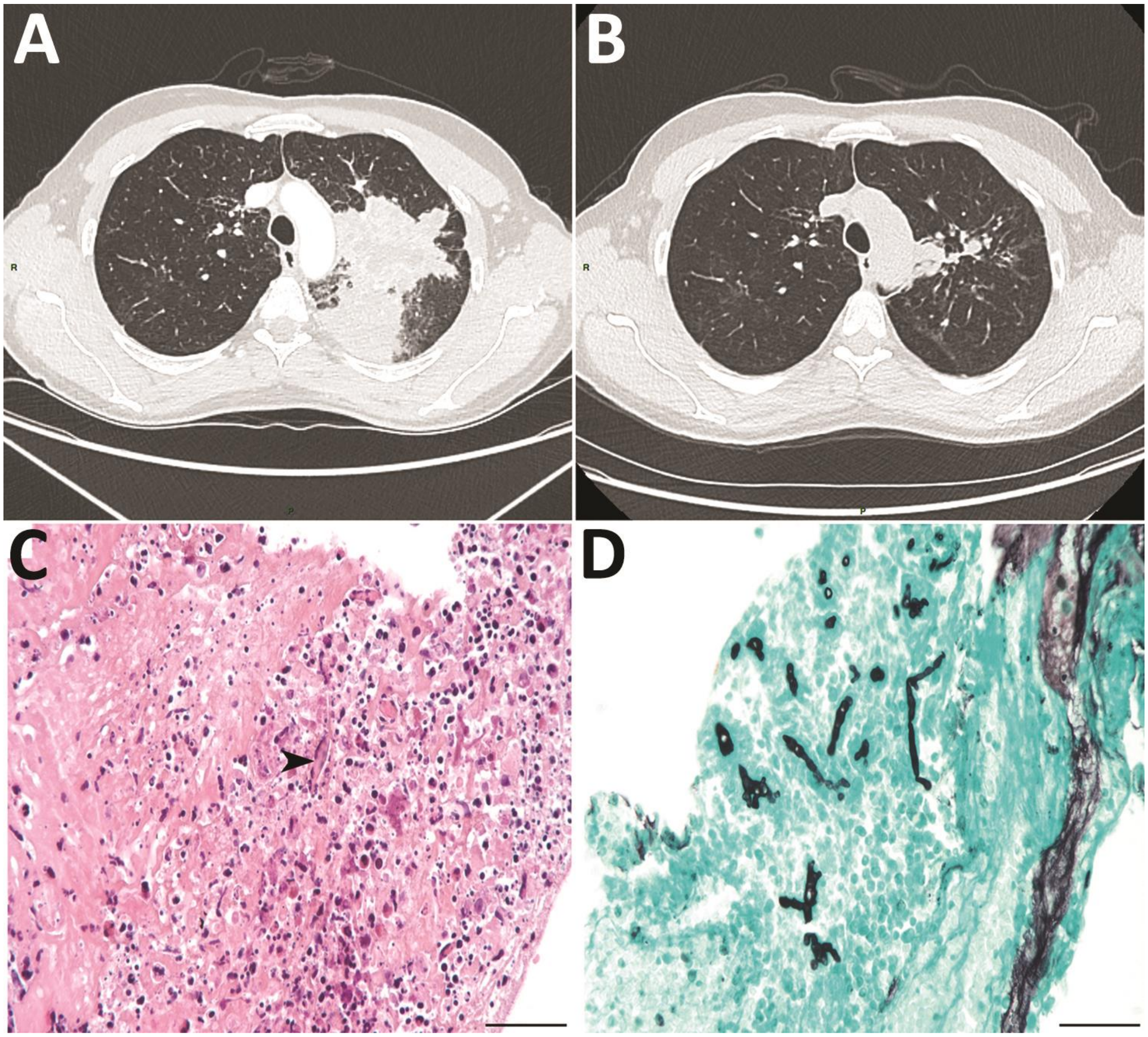

Appendix Figure. Images from a patient with chronic granulomatous disease who had *Aspergillus felis* infection. A) Thin-section (1 mm collimation) CT scan images at admission obtained at the level of the aortic arch showing a parenchymal consolidation of the upper-left lobe. B) Thin-section (1 mm collimation) CT scan images obtained 1 year after initiation of antifungal therapy. Samples of transbronchial biopsy specimens from the patient with *A. felis* infection were sent for pathological analyses. C) Hematoxylin and

eosin staining highlighting multifocal inflammatory lesions, centered on the chorion of a bronchus/bronchiole, with mixed inflammatory infiltrates containing numerous eosinophils and acute necrosis containing Charcot-Leyden crystals (arrowhead) (original magnification 400×). D) Gomori Grocott staining highlighting thin hyaline filamentous fungi with branching, septation, and multifocal distention. The small size of the biopsy did not allow us to adequately assess angioinvasion, but the fungi displayed local aggressiveness with invasion of the respiratory mucosa and chorion (original magnification 400×).
